# Artificial intelligence and science of patient input: a perspective from people with multiple sclerosis

**DOI:** 10.3389/fimmu.2025.1487709

**Published:** 2025-02-17

**Authors:** Anne Helme, Dipak Kalra, Giampaolo Brichetto, Guy Peryer, Patrick Vermersch, Helga Weiland, Angela White, Paola Zaratin

**Affiliations:** ^1^ Multiple Sclerosis International Federation, London, United Kingdom; ^2^ Dept. Medical Informatics & Statistics, The European Institute for Innovation through Health Data, Ghent University Hospital, Gent, Belgium; ^3^ Research Department, Italian Multiple Sclerosis Foundation, Genoa, Italy; ^4^ Multiple Sclerosis Society UK, London, United Kingdom; ^5^ Univ. Lille, Inserm U1172 LilNCog, Centre Hospitalier Universitaire de Lille (CHU) Lille, Fédératif Hospitalo-Universitaire (FHU) Precise, Lille, France; ^6^ Multiple Sclerosis South Africa, Western Cape, South Africa; ^7^ National Multiple Sclerosis Society, New York, NY, United States

**Keywords:** artificial intelligence, patient reported outcomes, health outcomes, multiple sclerosis, ethics

## Abstract

Artificial intelligence (AI) can play a vital role in achieving a shift towards predictive, preventive, and personalized medicine, provided we are guided by the science with and of patient input. Patient-reported outcome measures (PROMs) represent a unique opportunity to capture experiential knowledge from people living with health conditions and make it scientifically relevant for all other stakeholders. Despite this, there is limited uptake of the use of standardized outcomes including PROMs within the research and healthcare system. This perspective article discusses the challenges of using PROMs at scale, with a focus on multiple sclerosis. AI approaches can enable learning health systems that improve the quality of care by examining the care health systems presently give, as well as accelerating research and innovation. However, we argue that it is crucial that advances in AI – whether relating to research, clinical practice or health systems policy – are not developed in isolation and implemented ‘to’ people, but in collaboration ‘with’ them. This implementation of science with patient input, which is at the heart of the Global PROs for Multiple Sclerosis (PROMS) Initiative, will ensure that we maximize the potential benefits of AI for people with MS, whilst avoiding unintended consequences.

## 1 Introduction

There is an increasing demand for a shift towards predictive, preventive, and personalized medicine (1, 2) and artificial intelligence (AI) can play a vital role in achieving this. Multiple sclerosis (MS), an autoimmune condition affecting nearly 3 million people across the world (3), is very heterogeneous, affecting people’s lives in different ways. A single treatment or care approach will not be suitable for every individual. The presentation and course of MS reflect myriad factors that can be difficult to capture in a comprehensive manner. So, whilst MS is not itself particularly rare, once people with MS (pwMS) are sub-divided into groups requiring different treatment and care services, and who have different priorities when it comes to health outcomes, everyone becomes part of a rare group. Determining the right approach to treatment and care needs to take into account all of the variability that exists within that person’s life: their sex, age, environment, access to care, economic resources, comorbidities and many other factors. AI-based solutions may be necessary to support the capture and use of these complex data, so that health outcomes can be optimized for everyone.

## 2 Health outcomes that matter to people with MS

Health outcomes reflect information about the impact on people from health and care interventions. Leveraging patient experiential knowledge and make it scientifically measurable via Patient Generated Health Data (PGHD) is a critical part of the humanisation of health in line with Value-Based Health Care EU pillars (4–6). PGHD include patient reported outcome measures (PROMs), patient-reported experience measures (PREMs - people’s perspectives of their experience while receiving care) or Patient Preferences and Acceptability for Innovative health interventions (PPI). Among these, PROMs provide a patient perspective on the impact that a disease (and its treatment) has on their physical, functional, and psychological status without interpretation from anyone else. There is no unique definition of PROs: “any report of the status of a patient’s health condition that comes directly from the patient, without interpretation of the patient’s response by a clinician or anyone else” in accordance to the Food and Drug Administration (FDA) (7) or “any outcome evaluated directly by the patient him/herself and based on patient’s perception of a disease and its treatment(s)” in accordance to the European Medicines Agency (EMA) (8). The FDA definition of PROs designates both active and passive information as PROs, while the EMA definition seems to restrict PROs to active reports only. AI could help to incorporate PROMs reflecting different functional domains alongside other research and clinical data if relevant PROMs for the target population and adequate infrastructure for collecting PROs are available.

The Global Patient Reported Outcomes for MS (PROMS) Initiative launched on 12 September 2019 at the 35th Congress of the European Committee for Treatment and Research in Multiple Sclerosis (ECTRIMS). It is jointly led by the European Charcot Foundation (ECF) and the Multiple Sclerosis International Federation (MSIF) with the Italian MS Society acting as lead agency for and on behalf of the global MSIF movement (9, 10). The strategic intent of the PROMS Initiative is to engage people with MS in developing and prioritizing PROMs that give us a picture of their status today and changes over time. At present, clinical and care measurements are snapshots of individual functional domains and pwMS are frustrated that functional domains and corresponding interrelationships that matter most to them are not addressed by currently available PROMs (11). Within this framework, applying AI to PROMs can be a catalyst for a renewed humanism from research to care, but this vision will only be achieved by furthering the optimal engagement of pwMS (12).

## 3 The route to a unified view on PROMs for MS

Challenges with capturing, measuring and using PROs have been recently described by the PROMS Initiative (5) and are summarized below:

- reaching consensus on relevant PROMs for specific and targeted populations (i.e. acknowledging there cannot be a ‘one-size-fits-all’ approach for PROMs), which have been validated and can be used within and across countries for accurate comparisons;
- developing practical and usable tools (e.g. apps, wearables, other devices) to enable the routine capture of multiple changing outcomes over time, which requires acceptability and therefore a user-friendly and useful solution for collecting the information (13, 14);
- translating subjective impressions from PRO questionnaires (such as Likert scales) into valid numerical data, and determining what threshold constitutes a meaningful change for different individuals (15);
- calibrating changes in outcomes over time against the types and costs of health and care interventions that have created those outcomes. This can help target health spending most effectively (i.e. assessing value), without leading to unintended consequences such as restriction of access to care, support, disability status or benefits.

Commonly used PROMs in the MS field include the MS Impact Scale-29 (16), Multiple Sclerosis Quality of Life-54 (17), Patient Determined Disease Steps (18), SymptoMScreen (19) among others. At the current time, PROMs are mainly used as a correlate with classical metrics (in the case of MS, such as the Expanded Disability Status Scale (EDSS), Timed 25-foot Walk (T25W) and others). PROMs are used as confirmation of these classical metrics, rather than adding their own specific and unique value.

As mentioned earlier, pwMS are frustrated that currently available measures do not capture the experiences that have the greatest impact on their daily lives. In addition, PROMs also need to be measured formally so they can be collected consistently and compared over time for the same person and between people (20). There are many initiatives and resources focused on the creation and standardization of health outcome measures, including PROMs, for example the International Consortium for Health Outcomes Measurement (ICHOM) (21), the Patient-Reported Outcomes Measurement and Information System (PROMIS) (22), and the Core Outcome Measures in Effectiveness Trials (COMET) initiative (23). PROMOPROMS is an initiative focused on PROMs that matter most to people with MS and the implementation of these in clinical practice (24), and a recent global survey of pwMS identified the functional domains that have the greatest impact on their lives (25). Identifying distinct clusters of PwMS who share symptom patterns across functional domains and experiential knowledge, along with their interdependencies, will pave the way for a personalized application of PROMs from clinical trials to clinical practice and vice versa.

Despite this, there is limited uptake of the use of PROMs within the research and healthcare system. Without a significant body of evidence, health systems are poorly placed to learn, potentially ineffective interventions are sustained and health system budgets are wasted (26). The opportunity is also lost for PROMs to be used directly by people and their clinicians (27). The application of AI to PROMs data can support learning health systems, but a renewed humanism from research to care will only be achieved if researchers and the clinical community works effectively alongside people with MS.

The ALAMEDA project (28) made progress towards AI-enabled prediction, prevention and intervention. ALAMEDA is a Horizon 2020 EU-funded project aiming to make use of AI to reduce the costs of treating disorders such as MS, Parkinson’s, and stroke, hence easing the burden on healthcare systems. In a pilot study carried out by the Italian MS Foundation (FISM), wearable technology and smartphone apps enabled the longitudinal collection of continuous digital-health data and electronic PRO data from pwMS across domains including mobility, sleep, mental and cognitive ability, emotional status and quality of life. This data supported the development and testing of AI algorithms with the aim of detecting and predicting relevant changes in disease progression.

In particular, the MS pilot focuses on key aspects such as the use of predictive systems to improve decision support systems for multiple sclerosis and the use of wearable technology (from sensors to electronic patient reported outcomes) in MS. The end goal of the MS pilot study was to test AI/machine-learning based algorithms that are able to predict the risk of developing a relapse in MS. Therefore, a characteristic research interest of the MS study is to explore the use of combined PRO and wearable-provided data as input for relapse prediction algorithms (29).

Crucial to the success of the ALAMEDA project is the use of MULTI-ACT guidelines (30) to engage relevant and representative stakeholders, including pwMS. Through co-design with pwMS, preferences and opinions about devices, frequency of measurement and potential barriers and facilitators for adhering to long-term patient-reported data collection were identified. In addition, pwMS were also involved in identifying and prioritizing suitable endpoints that might act as signs of a forthcoming relapse. All these factors helped shape the final protocol for the ALAMEDA MS pilot study (29).

## 4 The potential for AI to improve health outcomes for people with MS

The use of AI within healthcare systems is not yet standardized or routine, and more research is needed into its cost-effectiveness. It includes interventions used by healthcare professionals such as AI-assisted clinical decision support systems, as well as those used by individuals, such as chatbots that provide health information and smartphones with AI-related applications. Applying AI technology to the analysis and use of health data – particularly when it has been patient generated or patient-reported - has the potential to improve prognosis, prevent and treat disease progression and improve lives, through taking a personalized approach to diagnosis, treatment and care (31, 32).

The role of AI in healthcare spans all clinical conditions and is widely studied, for example in the oncology field recent studies have examined whether machine learning models include PRO data, and how AI could impact the doctor-patient relationship (33, 34). In the field of MS, an example of a decision support system in development is ‘Clinical impact through AI-assisted MS care’ (CLAIMS), an AI-driven clinical decision-support platform that aims to model expected disease trajectories depending on treatment regimen (35). A review by Inojosa et al. (36) explores the opportunities for using large language models as a form of AI in MS management.

Crucially, the involvement of AI in research and healthcare must be guided by the science *with* and *of* patient input. The power of science *with* patient input relies on an innovative framework used to engage patients (10, 30), while the science *of* patient input relies on patient-generated health data (PGHD). Among PGHD, PROMs represent a unique opportunity to capture experiential knowledge from people living with health conditions and make it scientifically relevant for all other stakeholders – the mission of the Global PROMS Initiative (10).

With the advent of the European Health Data Space (EHDS), all EU member states will be required to focus on the quality and interoperability of priority health data items (37). The EHDS will enable large, enriched datasets encompassing information from the whole of the EU. Where standardized PROMs are in use for certain health conditions, collected in a clinical setting and stored in people’s medical records, these too will be available. The scale and complexity of data within the EHDS will necessitate the use of AI to interrogate these large datasets, combining clinical and PRO data to develop meaningful insights. AI will be instrumental in enabling greater use of PROMs in value-based healthcare decisions, such as those made by national health technology agencies, leading to improved delivery of healthcare across the region and better outcomes for individuals.

As set out in the framework by Rivera et al. (31), patient reported outcomes could be used as an input to an AI model, they could be an output predicted by the model, or an outcome in terms of the evaluation of the AI intervention. Within a healthcare setting, PRO measures may be used to monitor symptoms, monitor adherence to treatment, measure response to treatment, or determine when someone needs a clinical review. Using PROs in an AI or learning system could enable clinical decision making to incorporate the consideration of a person’s wellbeing, beyond overall survival or delayed progression of disease.

An example of how combining PROMs and AI could provide benefits for pwMS is through using AI approaches to interrogate individual-level data captured from multiple sources. PROs might be captured passively (e.g. via a smartphone enabled with technology such as a step-counter, accelerometer, altimeter etc) or input actively from a person recording their symptoms, feelings, use of medications and lifestyle factors such as diet and exercise. Added to this might be daily temperature or atmospheric pressure readings. PwMS report that fatigue is a huge challenge to daily living. Patterns uncovered by AI interrogation of complex patient-reported data over time could provide insights into which factors increase or decrease levels of fatigue. These factors could be environmental or aspects that can be influenced by the person through lifestyle changes or self-management. Importantly, if the AI model identifies consistent changes in data patterns over time, this might signal an underlying change in the condition, such as progression of MS, prompting referral to a healthcare professional.

## 5 Challenges with using AI in MS healthcare: perspective from people with MS

The increasing use of digital technology that deploys AI poses several challenges, including representativeness, data privacy, health equity and consent (38). When developing models or interventions involving AI and PRO data, an essential consideration is that the data used to develop and train AI systems needs to be representative of the population in which the AI approach will be implemented. If models are developed on a specific, limited population of people with a particular condition, there may be issues when applying them to people with different demographic backgrounds (39), which could lead to misdiagnosis or incorrect management. This is especially true for complex conditions such as MS, which can present very differently across individuals, especially when considered in the context of multimorbidity and on a global basis. In addition, a common symptom of MS is cognitive dysfunction. If a person is not able to provide PRO data that accurately reflects their condition, because the questionnaire is too complex for example, then the resulting dataset on which an AI model is trained may not reflect the real needs of the population.

Health interventions that involve AI will only make it successfully into the clinic if they are fully acceptable by people with health conditions and their clinicians and care providers. Trust and honest communication are crucial components of the interaction between a healthcare professional and a person with MS. Whilst there may be improvements to health outcomes from AI in terms of clinical decision making – and the latest AI technology developed by Google has even been shown to conduct sophisticated diagnostic conversations (40) - there could be a risk that overreliance on AI algorithms reduces a clinician’s ability to relate to people they are caring for as individuals. People want to see that their healthcare professional is also drawing on their experience and intuition as part of the decision-making process. Artificial intelligence might complement the role of healthcare professionals, but should not replace them.

A study comparing responses to frequently asked questions showed that people with MS rated those written by ChatGPT as higher in empathy compared by those written by a neurologist (41). Yet some people will find it hard to trust decisions that are purely an output of an AI system and any errors caused by use of such technology will have a profound impact on the relationship between a person and their clinician. McCradden et al. (42) argue that where health settings use AI-based predictions, these should not be prioritized above patient experiential knowledge. To enhance trust, people should be made aware when AI or algorithms are being used in decision-making relating to their healthcare. There needs to be transparency in terms of the data and instruments upon which AI and its underlying algorithms are based as well as any unconscious biases that may be inherent in both programming and interpretation. To help overcome barriers to uptake of AI health technologies, clinical trials of the technology should be co-designed with people with lived experience, and use relevant PROMs as a trial endpoint (43).

MS is a condition present across the globe. AI should not just improve outcomes for people with MS in well-resourced settings, and it is clear that AI has the potential to both improve and decrease health equity (44–46). In terms of MS healthcare, remote monitoring and digital technology that deploys AI algorithms could help fulfil a need caused by a lack of specialist healthcare professionals in some settings. If AI can improve the accuracy and speed of diagnosis, allowing for earlier intervention and personalized care plans, this should reduce the variation in care experienced by pwMS, both within and between countries. Yet the benefits of AI-assisted technology may not be available to everyone. The accessibility and costs of the technology – including any supporting infrastructure, personnel or regulatory requirements needed to integrate AI systems into the current system - may provide a barrier for lower socioeconomic populations (47) or countries where MS is relatively rare. A lack of use of the technology in these settings can contribute to a negative feedback loop, whereby the continual refinement and updating of the AI algorithms are based on a limited population, becoming increasingly less representative of the diversity of people with MS across the world.

A critically important consideration relates to privacy and security of personal health data. Whether in a clinical or research setting, the use of AI is likely to involve the collection and analysing of sensitive information. Also, personal health data may have social, cultural, and religious implications in communities that are less familiar with or accepting of health conditions such as MS. It is essential that safeguards are in place for handling, storing and using this type of data securely. People must have a clear understanding of the purpose for which their data might be used and give consent for their data to be used in this way. A focus on consent is even more important for people who may be experiencing cognitive dysfunction. It is important to remember, too, that data generated by and collected with AI and/or algorithms may produce consequences outside of health systems, including decisions regarding pensions, disability payments, and other services. For people with MS who rely on access to treatment, therapy, and other forms of support, there is a constant concern about the potential that this support could be restricted based on incorrect interpretation of personal data, whether by human or AI decision-making.

## 6 Discussion

How can we maximize the potential benefits of AI for people with MS, whilst avoiding unintended consequences? As mentioned earlier, this requires science with patient input, which is at the heart of the Global PROMS Initiative. Advances in AI – whether relating to research, clinical practice or health systems policy - should not be developed in isolation and implemented ‘to’ people, but in collaboration ‘with’ them. Underlying this, communication and transparency is key. Encouragingly, these considerations are reflected in the recent WHO guidance on the “Ethics and governance of artificial intelligence for health: guidance on large multi-modal models.” (48)

Quality of life is defined differently for everyone with MS and cannot be viewed purely clinically. AI algorithms cannot replace the emotional and psychological understanding of an individual and their expectations in relation to their wellbeing. The clinical interaction should always be ‘personal’, and it is important to guard against anything that reduces people to data points. There is a need for future research to determine whether AI in complement with standard of care has a beneficial impact on outcomes such as disability and quality of life.

As a community of people with MS, we urge that the use of AI in patient care proceeds with caution as well as anticipation. For care to maximize quality of life, it must be holistic, encompassing emotional, psychological and social as well as physical aspects. Any benefits from AI must not come at the expense of damage to the relationship between clinicians and the people they care for, widening health inequity, or worsening health and social outcomes for people with MS.

Crucially, the Global PROMS Initiative will help ensure that people with MS are involved in the development of PROMs for MS from research through to global implementation. They will have space to raise ethical questions in relation to the growing use of AI as it applies to large, patient-reported datasets. They can prompt other members of this multi-stakeholder initiative to move away from thinking of people with MS as data points, and consider the impact of any recommendations on all aspects of the life of a person with MS. Only by working collaboratively in this way will we ensure that future advances in AI safeguard individuals and be acceptable to the whole community.

## Data Availability

The original contributions presented in the study are included in the article/supplementary material. Further inquiries can be directed to the corresponding author.
